# Big Fish in a Big Pond: a study of academic self concept in first year medical students

**DOI:** 10.1186/1472-6920-11-48

**Published:** 2011-07-27

**Authors:** Kirsty Jackman, Ian G Wilson, Marjorie Seaton, Rhonda G Craven

**Affiliations:** 1 School of Psychology, University of Western Sydney, Locked Bag 1797, Penrith NSW 2571, Australia; 2 School of Medicine, University of Western Sydney, Locked Bag 1797, Penrith NSW 2571, Australia; 3 Educational Excellence and Equity Research Program, University of Western Sydney, Locked Bag 1797, Penrith NSW 2571, Australia

## Abstract

**Background:**

Big-fish-little-pond effect (BFLPE) research has demonstrated that students in high-ability environments have lower academic self-concepts than equally able students in low-ability settings. Research has shown low academic self-concepts to be associated with negative educational outcomes. Social comparison processes have been implicated as fundamental to the BFLPE.

**Methods:**

Twenty first-year students in an Australian medical school completed a survey that included academic self-concept and social comparison measures, before and after their first written assessments. Focus groups were also conducted with a separate group of students to explore students' perceptions of competence, the medical school environment, and social comparison processes.

**Results:**

The quantitative study did not reveal any changes in academic self-concept or self-evaluation. The qualitative study suggested that the attributions that students used when discussing performance were those that have been demonstrated to negatively affect self-concept. Students reported that the environment was slightly competitive and they used social comparison to evaluate their performance.

**Conclusions:**

Although the BFLPE was not evident in the quantitative study, results from the qualitative study suggest that the BFLPE might be operating In that students were using attributions that are associated with lower self-concepts, the environment was slightly competitive, and social comparisons were used for evaluation.

## Background

Over the past 25 years, research has demonstrated that equally able students have lower academic self-concepts in schools where the average achievement level is higher than in schools where the average achievement level is lower [1-3]. Known as the Big-Fish-Little-Pond Effect (BFLPE), this finding has been replicated in primary schools [4], high schools [2], and across countries and cultures [5,6]. Although the BFLPE has also been shown to have an effect on admission to elite universities [7], to the knowledge of the authors it has not been explicitly tested at the university level. The purpose of the present investigation was to explore whether the BFLPE [1,2,8,9] could be extended to medical students.

### What is Self-Concept and Why is it Important?

Self-concept can be defined as "a person's sense of self shaped through interaction with the environment and other people" [10]. A positive self-concept is regarded as important for good mental health, improving academic achievement [11,12], protecting against becoming a victim of bullying [13], and is seen as a key aim of education [14]. Although originally considered to be a unidimensional construct, Shavelson, Hubner, and Stanton [10] theorised that self-concept was multidimensional and hierarchically organised, with a global general self-concept at the apex and then split into two broader domains: academic self-concept [e.g. verbal, science] and non-academic [e.g. social, emotional]. Marsh and Shavelson [15] further developed this model by splitting the academic portion into two specific domains: verbal self-concept and mathematics self-concept. Research has since documented the multidimensional nature and the domain specificity of self-concept in academic [9,16], art [17], and sport [18] settings.

The BFLPE is specific to academic self-concept, a construct that refers to an individual's knowledge and perception of his or her level of competence or ability within the academic realm [19]. Research has shown that one's level of academic self-concept can influence factors such as course selection, long-term educational aspirations, educational attainment, academic attainment, and academic achievement [5,12,20,21]. For example, Phillips [22] showed that among equally able students, those with a low academic self-concept were portrayed by their teachers as lacking in persistence. In addition Marsh [2] showed that the higher a student's academic self-concept, the more likely it was that the student intended to attend university. Furthermore, in a ten-year study, Guay, Larose and Boivin [20] demonstrated that a positive academic self-concept was associated with better educational outcomes. Interestingly, Marsh and colleagues [4,23] have demonstrated that when highly successful students leave their regular academic settings and enter high ability settings, their self-concept declines.

### Self-Concept and Self-Efficacy

Self-concept has been considered to be fundamentally similar to self-efficacy. Self efficacy is defined as a person's belief that he/she has the ability to succeed in a specific task. However, these two constructs differ in various ways [24]. Self-concept judgements require evaluations of skills and abilities, whereas self-efficacy judgements concentrate on what people believe they can do with the skills and abilities they have. Additionally, while academic self-concept focuses on the past, academic self-efficacy looks forward to the future, and what an individual believes he/she could do. Furthermore, self-concept measures directly imply frame of reference effects, whereas self-efficacy measures do not. In judging one's academic self-concept the achievements of classmates are used as reference points, but no such reference points are implicated in self-efficacy items [25].

### Self-Concept and Attributional Theory

Weiner [26] located causal attributions for success and failure within three dimensions: locus, stability, and controllability. Locus refers to whether the cause is located within the individual (internal) or outside the individual (external). Stability refers to whether the cause is constant (e.g., ability) or temporary (e.g., luck). Some causes are controllable, such as effort, but others are not (e.g., luck [26]). According to Weiner, a causal attribution that is internal, stable, and uncontrollable, such as ability, will result in lowered self-concept and motivation; a causal attribution that is internal, unstable, but controllable, such as effort, will also reduce self-concept, but will increase motivation. Moreover, there is a considerable literature documenting what has been termed the self-serving bias [27], whereby individuals credit their successes to internal factors, but their failures to external factors. For example two studies [28] demonstrated that college students attributed academic success to causes that were more internal, stable and controllable.

In exploring the relation between attributions and self-concept Marsh, Cairns, Relich, Barnes, and Debus [29] found that students who attributed their successes and their failures to external causes were more likely to have lower self-concepts. These authors also demonstrated that attributing failure to a lack of effort was associated with lower maths, reading and general school self-concepts. Additionally, in a seminal work, Craven, Marsh, and Debus [30] employed a sophisticated feedback intervention with primary aged students that combined internally focussed performance feedback with attributional feedback. Their results suggested that "an increase in effort attributions in failure situations may conflict with a high self-concept".[p. 25] These findings are consistent with Weiner's [26] assertion that attributing failure to lack effort - an internal, unstable, and controllable attribution - results in lower self-concepts. Taken together the literature suggests that lower self-concepts are associated with external attributions and with internal attributions that, depending on the attribution, are stable or unstable, controllable or uncontrollable.

### Research Evidence for the BFLPE

According to the theoretical model underpinning the BFLPE, individual ability is positively related to academic self-concept ("I do well academically, so I feel good about my abilities"), but the average ability of the class or school is negatively related to academic self-concept ("There are students in my class (or school) who are really bright, and I don't feel as bright as them"). The BFLPE is characterised by this latter negative relation. Hence, the BFLPE is a positive outcome for students who are not included in elite classes or schools; they remain a big fish in a very small pond. However, for those students who do attend elite classes or schools, the outcomes are not so positive.

Research evidence for the BFLPE is considerable. It has been shown to exist at different levels of education [2,4-7], to have detrimental effects on educational outcomes (e.g., grade point average, educational and occupational aspirations, and the likelihood of taking advanced English and math classes [2];), and to be long-lasting. Marsh, Trautwein, Lüdtke and Koller [31] demonstrated that school-average achievement negatively predicted math self-concept two and four years after graduation from high school. The BFLPE also spans counties and cultures. In a study that demonstrated the BFLPE's universality, the BFLPE was shown to exist in 41 countries that were culturally and economically diverse [6]. The BFLPE is also extremely robust. In an evaluation of 16 potential BFLPE moderators (e.g., socio-economic status, motivation, self-efficacy, study methods, and behaviour) only three were found to moderate the BFLPE [32]. The BFLPE was greater for highly anxious students, for those who used surface learning as a method of self-regulation, or for those who had a preference for learning cooperatively. These authors concluded that their findings offered "support for the generalizability of the BFLPE and suggest that students are more similar than different in relation to the BFLPE" (p. 36).

### The BFLPE and Implications for Achievement

Based on a reciprocal effects model [REM], Marsh and Craven [21] emphasised that academic self-concept and academic achievement are reciprocally related, in such a way that high academic achievement is related to improvements in academic self-concept, and in turn high academic self-concept is related to improvements in academic achievement. In one of the first studies to test this model, Marsh and Yeung [33] assessed students' academic achievement and academic self-concepts across three years. Prior academic achievement was a statistically significant positive predictor of subsequent academic self-concepts for math, English, and science. When individual ability was controlled for, prior self-concept was a statistically significant positive predictor of subsequent math achievement. A similar, but weaker, relation was demonstrated for science and English. Hence, predictions from the REM were clearly supported by this study. The implication from this model is that for students to reach their full potential, both achievement and academic self-concept should be improved simultaneously. As demonstrated by BFLPE research, however, when students are segregated on the basis of their academic ability their academic self-concepts suffer, and according to predictions from the REM, their academic performance may also decline.

### The BFLPE and Social Comparison

Although they may be unaware of the term, people frequently engage in social comparison. They compare themselves with others in all sorts of ways, from comparing their salary packages, to their attractiveness and tennis ability. Marsh, Seaton, Trautwein, Lüdtke, Hau, O'Mara and Craven [34] have suggested that social comparison lies at the heart of the BFLPE. They have theorised that "students use the average level of academic accomplishments of other students within their school to form a frame of reference against which to evaluate their own academic accomplishments" [p. 326]. Empirical evidence for this was provided by Huguet, Dumas, Marsh, Regner, Wheeler, Suls, Seaton and Nezlek [35] who demonstrated that the BFLPE was based on comparisons that students made with the class as a whole. Hence, each class or school provides a particular frame-of-reference that students use for evaluation. As a result, "equally able students will evaluate their own academic accomplishments based on their current frame-of-reference, and this process will in turn affect academic self-concept" [36]. Thus, according to the BFLPE, it is better for academic self-concept to be a big fish in a little pond [to be a good student in a group of average ability students] than to be a small fish in a big pond [to be a good student in a group of high ability students]. Various suggestions have been proposed to overcome the BFLPE. For example, it has been suggested that reducing the amount of social comparison in the classroom by avoiding competitive environments may lessen the BFLPE [4].

### The Present Investigation

The present investigation consisted of two studies. Study 1 aimed to investigate quantitatively the academic self-concepts and social comparisons [measured by self-evaluations of ability compared with classmates] of first-year medical students to ascertain whether they declined during the year. Although we were unable to test for the BFLPE per se, as specific conditions need to be met to do so [34], we theorised that a decline in self-concept would be an indication that the BFLPE may be occurring. Being in a high-ability course [medical school] could negatively influence academic self-concept and self-evaluation of abilities as a result of being surrounded by high achieving peers (the BFLPE) [21], especially when performance is made salient in the form of examination results. In Year 1 the first opportunity that students get to compare their academic performance is after the midyear examinations when the results are released. In this study we measured academic self concept prior to the examinations and after. In Study 1, two hypotheses were formulated:

(1) Academic self-concept will be significantly lower at Time 2 compared with Time 1.

(2) Self-evaluations of ability will be significantly lower at Time 2 compared with Time 1.

Study 2 examined the relations between self-concept, social comparison, and academic achievement from a qualitative perspective. In relation to first-year medical students it aimed to: (1) gain an understanding of their thoughts and feelings about their individual academic performance; and (2) explore the nature of the peer group in the medical school environment. No hypotheses were postulated. Rather, research questions were formulated as follows:

(1) How do students perceive and evaluate their performance?

(2) Do students find the environment competitive?

Ethical approval was provided by the Universities Human Research Ethics Committee

## Study 1 - Method

### Participants

First-year medical students (*N *= 133) from an Australian university were invited to participate. The school is new with this cohort being the second cohort admitted. The course is a five year undergraduate program which takes school leavers, graduates of other degrees and applicants with partially completed degrees. The primary method of teaching in Years 1 and 2 is Problem-based learning tutorials. Year 1 is an integrated program which runs for the full academic year. Students sit a midyear examination consisting of three written papers. Results as a percentage score per paper are provided shortly after the examination.

### Measures

The Academic Self Description Questionnaire II (ASDQII) [8,37] was constructed specifically to measure academic self-concept. The six items used for each school subject were adapted by changing the subject name to "in most academic subjects". Items included "I am hopeless when it comes to most academic subjects". Students responded on an 8-point Likert type scale ranging from 1 (*strongly disagree*) to 8 (*strongly agree*). One negatively worded item was reverse scored. A higher rating meant that participants had higher academic self-concepts. The psychometric properties of the ASDQII have been shown to be acceptable [37].

Self-evaluation was used as a measure of social comparison. A single item, shown to be valid in previous social comparison research [38,39], was used. Participants were asked "How much better/worse are you academically compared with most of the other students in your year". Students responded on a 5-point Likert scale ranging from 1(*much worse*) to 5 (*much better*), with a mid-point of 3 (*the same*).

### Procedure

At both Time 1 (Semester 1) and Time 2 (Semester 2) the surveys were delivered to all first-year medical students during problem-based learning tutorials. Time 1 and Time 2 were both very early in the respective semester. Each student was provided with an information sheet, consent form and the survey and asked to return them to the tutor or to a box at school reception. Most chose to return their survey to the front desk so students were not aware of who was completing the survey. Students were able to put their name on the survey so that longitudinal analysis could occur.

Performance was made salient by administering the surveys at Time 2 after students had received their grades for Semester 1. However, due to a low response rate, the survey questions were also put on-line and participants were emailed a direct link to the survey. A total of 22 students completed the survey at Time 1 and of these 20 students also completed the Time 2 surveys.

### Statistical Analyses

Data were analysed using SPSS v.17. Alpha was set at .05. Assumptions of normality were found to be satisfactory for both academic self-concept and self-evaluation. A paired samples t-test with a two-tailed approach was used to compare results between Time 1 and Time 2.

## Study 1 - Results

Twenty students volunteered (13 female). Ages ranged from 17 to 38 years, with a mean age of 20.65 years (*SD *= 4.92). Cronbach's alpha for the ASDQII with the current sample was .92 at Time 1 (*M *= 6.48, *SD *= 0.95) and .79 at Time 2 (*M *= 6.56, *SD *= 0.69). (See Table 1).

**Table 1:** Mean and Standard Deviation scores for Academic Self-Concept and Self-Evaluation across Time

	Time 1		Time 2	
**Variables**	***M***	***SD***	***M***	***SD***

Academic Self-Concept	6.48	.95	6.57	.69
Self-Evaluation	3.50	.61	3.45	.76

Number of students = 20

Means for Self-evaluation scores at Times 1 and 2 are shown in Table 1. Self-evaluation at Time 1 and 2 and academic self-concept at Time 1 and 2 were moderately correlated (self-evaluation: *r *= .62; academic self-concept: *r *= .51). Self evaluation and academic self concept did not significantly correlate with each other at Time 1 or Time 2 (Time 1: *r *= .18 *p *= .44; Time 2: *r *= .32 *p *= .16). Self evaluation at Time 1 correlated moderately with academic self concept at Time 2 (*r *= .52 *p *= .02).

## Study 1 - Conclusions

Hypotheses 1 and 2 were not supported. Contrary to predictions, there was no statistically significant difference for academic self-concept between Time 1 and Time 2, *t *(19) = -0.498, *p *>.05. Additionally, again contrary to predictions, there was no significant change in self-evaluation between Time 1 and Time 2, *t *(19) = .326, *p *> .05. The results suggest that medical students' academic self-concepts and self-evaluations had not changed over time. Although students had knowledge of their academic performance from Semester 1 this knowledge did not seem to affect their academic self-concepts and self-evaluations at Time 2.

Academic self-concept is based, in part, on how one feels one has performed compared with others. So, the lack of correlation between academic self-concept and self-evaluation at Time 1 and again at Time 2 is surprising. At Time 1 perhaps this is understandable as self-evaluation was based on students' comparisons with other students in the year group and at Time 1 they would have had no basis for these comparisons. However, at Time 2 students should have had a basis for this comparison. Also, self-evaluation at Time 1 and academic self-concept at Time 2 were positively correlated. These findings are surprising and future research is needed to elucidate these issues.

Although medical students are in a highly selective environment, the effects of being within that setting and a part of that course had no effect on academic self-concept or on their self-evaluation. There may be several reasons for this. Firstly, only 20 of a total of 133 students participated. These students may not be representative of the total sample. They may be students who did well in the first semester and so their academic self-concepts and self-evaluations were not affected by grades. Apart from obtaining a more representative sample, future research should also take grades into account and use structural equation modelling to assess the causal relations between measures of achievement, academic self-concept, and self-evaluation. Secondly, the measures were taken at the beginning of both semesters and overall quite early within the course, so there could be a reflected glory or assimilation effect occurring. Marsh, Kong, and Hau [23] found that "higher school-average achievements led to lower academic self-concepts (contrast effect), whereas higher perceived school status has a counterbalancing positive effect on self-concept (reflected-glory, assimilation effect)" [p. 337]. It may be these first-year medical students are still feeling the glory of being selected into medical school.

## Study 2 - Method

### Participants

All first year medical students (*N *= 133) from the same Australian university as in Study 1 were invited, via email, to participate in scheduled focus groups. This recruitment was separate from that used in the first study. Twenty-six (13 male) students agreed to participate.

### Procedure

Author 1 conducted five semi-structured focus groups at the beginning of Semester 2. The number of students in each group was 4, 8, 7, 2, and 5, representing almost 20% of the student cohort. Each session was structured in the same way using a set agenda of questions. To fit within the student timetable sessions were held at lunchtime and food and drink were provided. Students signed consent forms and the sessions were digitally recorded, then transcribed verbatim with all identifying information removed.

### Analysis

Data were manually processed via an inductive thematic analysis procedure in which connections, themes, and thematic patterns were identified and explored within the data [40].

## Study 2 - Results

Almost 20% (n = 26) of the student cohort took part in the focus groups. The students were volunteers and that needs to be taken into consideration in understanding the results.

### Research Question 1. Perceptions and Evaluations of Academic Performance

This Research Question considered how medical students perceived their performance and how they evaluated it. Students had received results for Semester 1 examinations, so their performance was particularly salient to them. Information they received concerning the results was their own mark, the mean, maximum and minimum scores, and the 25^th ^and 75^th ^percentile scores.

Two minor themes to emerge concerned students who made no attributions concerning their performance. In the first one, students reported being satisfied with their results (5 responses; 20.8% of total responses; see Table 2):

**Table 2:** Perceptions of Performance

Themes	Number of Responses	Percentage of Total Responses in this Category
External Attributions	9	37.5%
Internal Attributions - Effort	7	29.2%
Satisfied [no attributions]	5	20.8%
Room for improvement [no attributions]	3	12.5%
TOTAL	24	100.0%

Number of interviewees = 26

"I'm actually pretty happy with my results. I'm getting quite good results and it's a nice sort of thing to know that I am able to maintain what I've done previously."

In the second minor theme, students felt that they could do better in future (3 responses; 12.5% of total responses; see Table 2):

"Comparatively it's good but personally I'd like to do better."

The two main themes to appear in the responses concerned internal and external attributions. In the main theme, students attributed their poor academic performance to external attributions. These included the examination questions being irrelevant, having other commitments that prevented them from studying, not knowing what to expect, and having a very bright peer group (9 responses; 37.5% of total responses; see Table 2):

"I thought I did put a lot of work into it but often I found lots of the questions, .... in the exam which for many of us who would have perceived it as irrelevant." 

"Yeah I spend a fair bit commuting to University each day, or driving. I spend maybe 3 ½ hours on the road each day, which is a pain. And yeah, as a result of doing that and plus a lot of volunteer work and being on call, I barely get any study done and plus other work commitments, my family's business which of course is feeling a lot of hardship given our global financial crisis. Yeah, it's hard."

"Because coming like, I came here straight out of high school, everything was sort of there for you. You could study all you needed and get through the exams and you'd be fine. But here, there's just so much things, you just can't really just study the things to answer questions, you have to study everything because they can ask you anything. And having that change, it sort of took a lot to get used to."

"... I think I'm surrounded by yeah, really academically bright people, like it's sort of, like overwhelming that sort of feeling."

Previous research has demonstrated that attributing failure to external causes was associated with slightly lower self-concepts [29]. The self-concepts of students in the present investigation who used external attributions may have been low, and this may have been due to receiving their examination results.

In the second main theme, students discussed the amount of effort that they felt they had exerted. These students felt that they could do better if they put in more effort - an internal attribution that is unstable but controllable. (7 responses; 29.2% of total responses; see Table 2):

"Results are reflective of the work I put in which was very little, I don't feel good about that but it's the way it is..."

"It was like lazy 'cause I'm a huge procrastinator, I didn't actually start studying for these exams until like a week before the exams, so you know like I know that I'm disappointed with the result, but I know that 'cause I didn't put the work in either."

Attributing failure in this way has been shown to have a negative relation with self-concept [29,30]. Perhaps the self-concepts of students using such internal attributions may have been low, and as before, this may have been due to having received their examination results. Low self-concepts may also be due to students evaluating their performance by comparing their results with those of others. When asked how they evaluated their performance, students stated that they used the statistics given to them to compare their results and evaluate their performance (8 responses in total):

"Cause like they did release the mean, the highest and lowest marks so like when you have that, you compare yourself."

"...because they posted the grades for each like quartile, each like group so I think that was a good way to compare ourselves to everyone without actually like seeing everyone's grades."

Students appeared to compare themselves with their classmates as a whole - perhaps because individual results were not presented. Previous research has shown that students do compare in this way [25], and that this type of comparison is associated with lower self-concepts and is the basis of the BFLPE.

### Research Question 2. Peer Group

This Research Question considered whether students felt their peer group was competitive. To be accepted into medical school in Australia, students need to have a high grade point average or have achieved very highly in the standardised tests that occur at the end of high school. As such, students in medical school tend to be highly intelligent. Research has demonstrated that moving into a situation populated by extremely intelligent others can be detrimental to one's self-concept [36] and result in the BFLPE. The approach within this particular University is away from competitiveness and more towards a cooperative environment. By minimising the competitiveness that perpetuates social comparison processes, it has been suggested that the BFLPE may be lessened [41]. With this in mind, students were asked to comment on whether the environment was competitive or not.

Forty-two percent of responses (8 responses; see Table 3) indicated that the environment was not competitive:

**Table 3:** Peer Group

Themes	Number of Responses	Percentage of Total Responses in this Category
Slightly Competitive	11	57.9%
Not Competitive	8	42.1%
TOTAL	19	100.0%

Number of interviewees = 26

"I think it's actually really nice it's not competitive, I've heard that other Med schools are a lot more competitive, like they'd actually know and they really don't help each other and stuff so."

"I don't think it's very competitive, I think a lot of people like help each other and like, you know, like people just post things on like CT and stuff to help each other out with PBLs and exam questions. So I think that yeah, I've never felt like people are very competitive."

Most responses (11 responses; 57.9%; see Table 3) indicated that it was a slightly competitive environment. Some said that they felt competitive so that they could keep up with the others; others felt that while some were competitive, others were not; and some felt that there were others who were unwilling to share information:

"..like if I see friends studying in the library I'm more compelled to study myself in the library rather than just do what I want to do so in that sense I'm a little bit competitive but I'm not trying to like beat them, I just don't want to fall behind I guess, so yeah."

"..coming here to medicine was like going straight back to high school, .....so some are competitive, some aren't"

"And what I found is a lot of, some of the school leavers from selected schools are particularly competitive. They went around at the end of last semester saying I've got all the past papers from Melbourne and they wouldn't share them."

One of the prominent themes found in the analysis concerning competitiveness was in relation to students' previous exposure to highly academic surroundings.

"I feel the same because like compared to my high school, everything is ...competitive it's just oh - 'cause at [name of school] it's not a good environment to become a proper person. Like it's good to study and stuff ...Yeah. So it's just like when I come here there's not a lot of people that are more competitive to where I previously went yeah, at [name of school] it's like that you can't become a proper person there. Here it's like people take like it more easy, people aren't so highly strung, ...."

"I don't think it's competitive but we all, sort of, try to help each other, I think, but on the inside we still are, sort of, competitive, seeing as a lot of us come from like selective schools and high schools where you, sort of, have to fight for your position, like - like you still, sort of, feel it a bit, I feel it a bit like the competitiveness, like sort of, on the inside so people like, you know, I just don't want to give this to that person 'cause they'll beat me. "

These comments suggest that prior exposure to a highly academic environment may act as a buffer against the transition into another highly academic setting. This may result in these students not feeling overwhelmed by their new environment. Consequently these students may be able to maintain their belief in their academic competence. Or, it may be that attending high-ability schools may already have undermined the perceptions of their competence - the BFLPE.

Although this medical school attempted to reduce competitiveness it does not seem to have eliminated it completely. Students reported finding the environment slightly competitive and, as already noted, students used social comparison to evaluate their performance. Competitive environments and comparing with others are contributing factors to the BFLPE. Hence, the focus group responses suggest that the BFLPE may be occurring.

## Conclusions

Social comparison and academic self-concept have been the topic of substantive investigation, but, most of this research has focused on school students within academically selective schools. This is the first study to consider these constructs within the University setting.

Although we were not able to show a reduction in self-concept in Study 1, results from Study 2 suggest that self-concept may not be as strong as it could be. Firstly, students were using attributional styles that have been associated with lower self-concepts. Secondly, even although this particular medical school espoused a cooperative environment, Study 2 results indicated that students found the environment slightly competitive and were using social comparison to evaluate their performance, both contributing factors to the BFLPE. Hence, there may have been a decline in self-concept in Study 1 [that would indicate the presence of the BFLPE] that we were unable to detect due to the small number of participants. Future research should be based on larger samples and include longitudinal designs.

The major strength of this study is in being the first of its kind to examine the BFLPE at the university level with medical students. In doing so, it is the first step in extending the BFLPE model from a school setting and into the tertiary realm. Another strength is in its multi-method design. There is growing recognition that quantitative and qualitative research methods complement each other in ways that both consolidate findings and shed light on issues that are best studied by one or the other [42]. The qualitative study was able to provide insights concerning the self-concepts of medical students that the quantitative study was unable to do.

Unfortunately, as both studies were based on a small sample from one medical school they are therefore of limited generalisability. Being a new school the students are subject to significant evaluative and research surveys and at the time of these surveys students were demonstrating significant survey fatigue. Future research could consider using a larger sample from multiple universities. The quantitative study was anonymous and it was impossible to link survey results with qualitative data. Future research could be designed to match qualitative and quantitative samples.

## Abbreviations

ASDQII: Academic Self Description Questionnaire II; BFLPE: Big Fish Little Pond Effect; PBL: Problem-based learning; SPSS: Statistical Package for the Social Sciences

## Competing interests

The authors declare that they have no competing interests.

## Authors' contributions

RC conceived of the research and the approach. KJ conducted the research under the supervision of MS and IW. MS and IW prepared the first drafts of the manuscript and all authors read and approved the final manuscript.

## Pre-publication history

The pre-publication history for this paper can be accessed here:

http://www.biomedcentral.com/1472-6920/11/48/prepub
